# Continuous Lighting Promotes Plant Growth, Light Conversion Efficiency, and Nutritional Quality of *Eruca vesicaria* (L.) Cav. in Controlled Environment With Minor Effects Due to Light Quality

**DOI:** 10.3389/fpls.2021.730119

**Published:** 2021-10-12

**Authors:** Simona Proietti, Stefano Moscatello, Francesca Riccio, Peter Downey, Alberto Battistelli

**Affiliations:** ^1^Consiglio Nazionale delle Ricerche, Istituto di Ricerca sugli Ecosistemi Terrestri, Porano, Italy; ^2^Department of Applied Science, Limerick Institute of Technology, Limerick, Ireland

**Keywords:** rocket, controlled environment agriculture (CEA), carbohydrate, fibers, nitrate

## Abstract

Light-emitting diode lamps can allow for the optimization of lighting conditions in artificial growing environments, with respect to light quality, quantity, and photoperiod extension, to precisely manage resources and crop performance. *Eruca vesicaria* (L.) Cav. was hydroponically cultured under three light treatments to investigate the effect on yield and nutritional properties of rocket plants. A treatment of (W-12h) having a12/12 h light/dark at 600 μmol m^−2^ s^−1^ provided by LEDs W:FR:R:B = 12:2:71:15 was compared with two treatments of continuous lighting (CL), 24 h light at 300 μmol m^−2^ s^−1^ provided by cool white LEDs (W-CL), and by LED R:B = 73:27 (RB-CL). CL enhanced the growth of the rocket plants: total fresh biomass, leaf fresh weight, and shoot/root ratio increased in W-CL, and leaf dry weight, leaf dry matter %, root fresh and dry weight, and specific leaf dry weight (SLDW) increased in RB-CL. Total carbon content was higher in RB-CL, whereas total nitrogen and proteins content increased in W-12h. Both W-CL and RB-CL increased carbohydrate content in the rocket leaves, while W-CL alone increased the sugar content in the roots. Fibers, pigments, antioxidant compounds, and malic acid were increased by CL regardless of the light spectrum applied. Nitrate was significantly reduced in the rocket leaves grown both in W-CL and RB-CL. Thus, the application of CL with low light intensity can increase the yield and quality value of rocket, highlighting that careful scheduling of light spectrum, intensity, and photoperiod can improve the performance of the crop.

## Introduction

Light is an essential environmental factor affecting plant growth, development, and phytochemical biosynthesis over short and long periods of growth as a result of the functionality exerted by radiation intensity and spectral composition. Plants exhibit high plasticity to variations in light characteristics either when using radiation as a source of energy for photosynthetic processes, or when it represents a signal to regulate photo-morphogenetic responses *via* a complex system of wavelength-specific photoreceptors (Paik and Huq, 2019; Paradiso and Proietti, 2021). The optimal light setting, in terms of light quantity and quality is a key element of controlled environment agriculture (CEA) where all factors are controlled to optimize productivity and resource use efficiency (Graamans et al., 2018).

Controlled environment agriculture technologies were introduced as early as the 1970s, in preparation for growing plants for food production during future NASA space missions (Gitelson et al., 1976). Today, CEA approaches are applied to plant production in contexts of climate changes scenarios, increasing population, extreme environmental conditions, unfavorable rural areas, and urban agriculture (Graamans et al., 2018). In advanced CEA plant growth facilities, light-emitting diodes (LEDs) have emerged as the most efficient and adaptable among artificial lighting systems. LEDs have many favorable characteristics when compared with previously available lamp types. Among them is their low energy requirement, low radiant heat output, very fast response time and tunability, long duration, and the availability of a large variety of narrowband-emitting diodes. This latter characteristic may allow for the scheduled induction of spectral-dependent physiological responses of plants, ensuring the optimal light setting for both crop yield and quality (Singh et al., 2015). However, the selection of optimal lighting conditions for the growth of different plant species is far from resolved. Extensive scientific literature highlights varying responses of plants to different light settings even when same light conditions are applied to different species (Paradiso and Proietti, 2021). Most of the studies conducted so far emphasize the role of red and blue wavelengths, when applied either individually or in a synergistic combination, due to the high absorption by photosynthetic pigments (Samuoliene et al., 2012; Wang et al., 2018). However, a more relevant role has been recognized recently for green light (Smith et al., 2017). It is clear then that a careful evaluation of the response to the light environment should be performed for each species as well as the growing conditions if maximizing efficiency, yield, and quality is the target.

Providing artificial light to growing plants is expensive; therefore, all solutions that increase the efficiency by which energy inputs are positively converted into yield and quality are relevant to the success of CEA technologies, both on Earth and, in the future, in space. One possible way to increase light efficiency in CEA systems is extending the duration of daily illumination period. The use of continuous light (CL) could provide plants with a high (and optimal) daily total photosynthetic photon flux density (PPFD) while reducing the quantity of photons applied per unit of time, the electric power peaks required by the system, and the size of both the illumination and cooling subsystems. This could be a very efficient way to manage light in CEA systems where the light flux is typically quite low, down to 10 times compared to open field conditions, with possible detrimental effects on both yield and quality.

Studies on plant cultivation in extended photoperiods show some potential benefits of 24-h CL for crop production in both greenhouses and closed controlled environment systems (Gitelson et al., 1976, Sysoeva et al., 2010; Velez-Ramirez et al., 2011). However, the use of CL on plant cultivation presents a challenge, because plant responses are contradictory and are far from being fully understood and modeled. Under a reduced value of PPFD and CL, some species accumulate more dry matter, starch, and soluble sugars, and higher amounts of different antioxidant metabolites (Haque et al., 2015). Changes in light quality and photoperiod can also affect the lignin content in wheat (Dong et al., 2015) and the cellulose synthase complex responsible for the modulation of cell wall components (Bischoff et al., 2011). CL may also have a detrimental effect on plant growth and yield. Evidence of this was shown in intolerant cultivars of potato, where the total growth was strongly reduced (Wheeler and Tibbitts, 1986), and in tomato, which developed leaf chlorosis rapidly after exposure to continuous light (Globig et al., 1997) lettuce (Kitaya et al., 1998), sweet pepper (Murage and Masuda, 1997), and kale (Lefsrud et al., 2006). Reasons for the reported differences in plant responses to CL are largely unknown (Velez-Ramirez et al., 2011; Haque et al., 2015).

*Eruca vesicaria* (L.) Cav., from the Brassicaceae family, represents an important food crop with a high commercial value, which is used as a fresh salad worldwide. Rocket leaves contain high concentrations of bioactive metabolites effective in promoting human health preservation (vitamins, antioxidants, carbohydrate, proteins, fibers, minerals, and secondary metabolites); however, it is also a potential source of unsafe nitrates that may be linked to organoleptic traits contributing to consumer preference and acceptance (Bell et al., 2017). The metabolome of the rocket might be strongly affected by the environmental conditions prevailing during plant growth and development (Riga et al., 2019). *E. vesicaria* was germinated on board the International Space Station (Colla et al., 2007); it was selected among the species suitable for food production in space (Dueck et al., 2016). It is known to be suitable for growth and production under fully controlled growing conditions on both Earth- and space-oriented systems (Jones-Baumgardt et al., 2020; Zabel et al., 2020). Our working hypothesis is that the efficiency of the cultivation of *E. vesicaria* under indoor conditions can be ameliorated by CL without detrimental effects on the yield and quality. Hence, we tested the yield and quality response of *E. vesicaria* to CL and different light recipes under fully controlled growth conditions. Our aims were to a) test the possibility of growing rocket under CL and whether there is a dependency on the light spectrum provided; b) ascertain if CL, in combination with specific light spectra, could increase light use efficiency of the rocket crop under fully controlled growing conditions with respect to a 12-h photoperiod; c) to verify if key quality characteristics of the rocket can be positively or negatively affected by a 24-h growth cycle and determine if the response is dependent on light spectrum and intensity.

## Materials and Methods

### Plant Materials and Growth Conditions

Rocket, *Eruca vesicaria* (L.) Cav., EDEN selection (La Semiorto Sementi, Sarno Salermo, Italy), was grown at the Consiglio Nazionale delle Ricerche-Istituto di Ricerca sugli Ecosistemi Terrestri (CNR-IRET) under fully controlled environmental conditions in a growth chamber (Fitotron SGD170 Sanyo; Gallenkamp, Leicestershire, United Kingdom) (Proietti et al., 2013). In this experiment, the chamber was equipped with two LED lamps (model LX60; Heliospectra AB, Gothenburg, Sweden). Two seeds per tube were sown in two 2 ml Eppendorf tubes (Eppendorf Srl, Milan, Italy), filled with perlite (Perlite Italiana Srl, Milan Italy) and whose bottom end was removed. The Eppendorf tubes were positioned in plastic racks (24 × 34 cm) each with 224 seats. Each rack was fixed on the top of a container (25 × 35 × 40 cm) with 8 L of water, with half of the tube immersed in water. Seeds were germinated under 150 μmol m^−2^ s^−1^ photosynthetically photon flux density (PPFD), provided for 12 h per day (12-h dark) by cool white 5700 K LED. One week after germination (time zero of the growth period), only one seedling was maintained in each seat. Two hundred twenty-four plants were grown in each container at a density of 2,740 plants per m^2^, and the water was substituted with an aerated and complete nutrient solution as the one selected for leafy plants in the EDEN ISS project (Zabel et al., 2020). The nutrient solution was renewed every week, ensuring that pH and EC were maintained at 6.5 and 1.7 mS cm^−1^, without relevant changes. A temperature of 22 ± 1 /25 ± 1°C night/day, relative air humidity of 70 ± 5%, and CO_2_ concentration of 600 ± 10 μmol mol^−1^ (ppm) were maintained.

### Light Treatments

**Figure 1:**
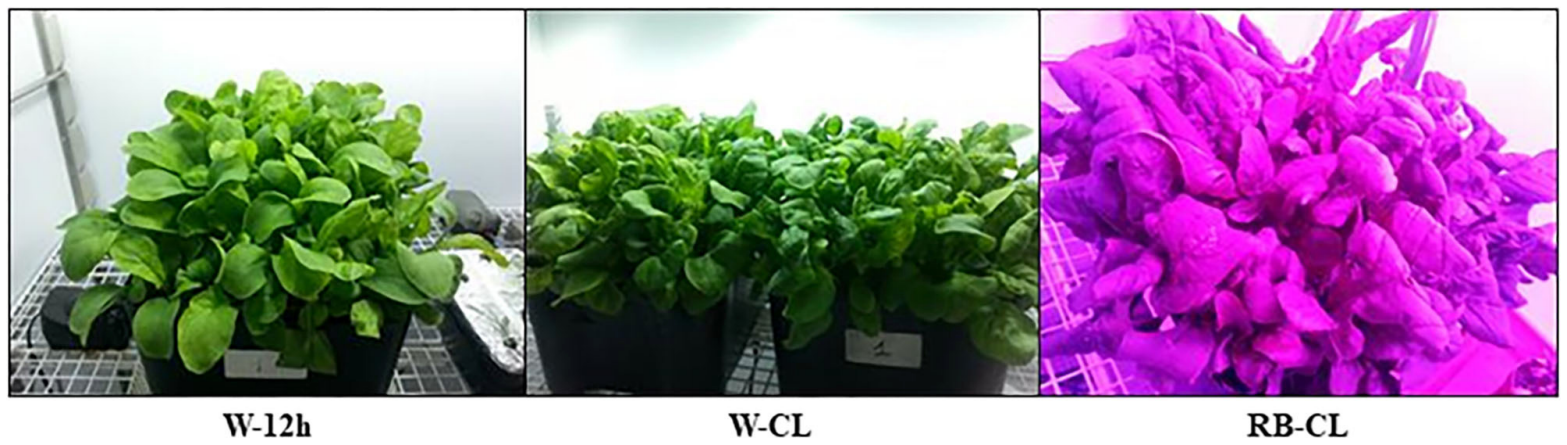
Morphological differences in *Eruca vesicaria* (L.) Cav. grown under three different light-emitting diode (LED) treatment conditions. Treatment 1—(W-12h): photoperiod of 12-h light/12-h dark; photosynthetic photon flux density (PPFD) was 600 μmol m^−2^ s^−1^, LEDs W:FR:R:B = 12:2:71:15. Treatment 2—(W-CL): photoperiod of 24 h continuous light (CL); PPFD was 300 μmol m^−2^ s^−1^, cool white LEDs. Treatment 3—(RB-CL): photoperiod of 24-h continuous light; PPFD was 300 μmol m^−2^ s^−1^, LEDs R:B = 73:27. All the light treatments received the same daylight integral (DLI) (25.92 mol quanta m^−2^ day^−1^).

The growth chambers are equipped with a transparent glass roof with LED lamps placed on the top in a fixed position, ensuring the most homogenous light distribution on a horizontal surface at 50 cm from the lamp output. As the plants grew in height, the lamp-to-plant canopy distance was adjusted by moving the plant tray inside the growth chamber to maintain the 50 cm lamp distance from the top of the canopy. The lamps were equipped with four types of LEDs, and each could be tuned on a relative scale (0–1,000) through a computer linked to a router. The emission characteristics of the different LED types were as follows: blue LEDs (B), 450 nm; red LEDs (R), 660 nm; far red LEDs (FR), 730 nm; and the cool white LEDs (W), 5700 K. The W LEDs had an emission spectrum of B = 26%, green (G) = 45%, R = 27%, and FR = 2%. Three light treatments were set from time zero of the growing period until harvest (Figure 1): Treatment 1 (W-12h) with a photoperiod of 12-h light/12-h dark and PPFD of 600 μmol m^−2^ s^−1^ provided by W:FR:R:B = 12:2:71:15; Treatment 2 (W-CL) with a photoperiod of 24-h continuous light and PPFD of 300 μmol m^−2^ s^−1^ provided entirely by cool white LEDs; and Treatment 3 (RB-CL) with a photoperiod of 24-h continuous light and PPFD of 300 μmol m^−2^ s^−1^ provided by R:B = 73:27. The PPFD of 600 μmol m^−2^ s^−1^ was selected, as it is the maximum that can be achieved by the light delivery system setup of the EDEN ISS project. All the light treatments received the same daily cumulative intensity of photosynthetically active quanta (as daylight integral – DLI = 25.92 mol m^−2^ day^−1^).

### Plant Measurements and Compositional Analysis

Harvest occurred 30 days after time zero. Half of the available plants were used for the determination of shoot and root fresh biomass weight (FW), shoot and root dry weight (DW), dry matter (DM) percentage, shoot/root (S/R) ratio, specific leaf dry weight (SLDW), total carbon (C) and nitrogen (N) percentage, and ash content. The plants were sampled randomly in different positions inside the growth chamber. The number of replicates for each determination is reported in the captions of the Tables. The drying of the plant material was performed by freeze-drying to constant weight. Leaf area was measured by a leaf area meter (LI-3100; LI-COR Biosciences, Lincoln, NE, United States). SLDW (dry weight/leaf area) was calculated using leaf disc of 1.88 cm^2^ cut from the lamina with a cork borer. The dried samples were reduced to a fine powder in a mill before analysis. Ash content was determined only in aerial parts of the plants by ignition of the dried powder at 575°C in a furnace (P 300; Nabertherm GmbH, Lilienthal, Germany), with temperature ramping according to the NREL/TP-510-42622 method (Santi et al., 2015). Carbon (C) and nitrogen (N) content of the leaves was analyzed using an elemental analyzer (Model NA 1500; Carlo Erba, Milan, Italy) and expressed as % of the dry matter. C:N molar ratio was determined as: C:N = (% C/12)/(% N/14). Total proteins content was obtained from the total N concentration corrected by subtracting the N due to nitrate ion and applying a conversion factor of 6.25 (Scartazza et al., 2017). For the quantification of non-structural carbohydrates (NSCs), antioxidants, pigments, organic acid, and inorganic anion concentration, fresh samples were collected and immediately frozen in liquid nitrogen, ground to a fine powder in a mortar using a pestle under liquid nitrogen conditions, and preserved at −80°C until analyses.

### Non-structural Carbohydrates

Measurements of NSCs were performed using 70 mg subsamples of the powder stored at −80°C. Extraction was performed in 1 ml of 80% ethanol at 80°C for 45 min under continuous shaking conditions. The extract was centrifuged at 16,000 × *g* for 5 min, soluble sugars (glucose, fructose, and sucrose) were recovered in the supernatant, and starch was in the pellet. Soluble sugar determination, by spectrophotometric coupled enzymatic assay, was performed, as described in Scartazza et al. (2017). The pellet, containing starch, was washed four times with a 50 mM NaAcetate buffer (pH 4.5) and then suspended and autoclaved at 120°C for 45 min in 1 ml of the same buffer. After autoclaving, the sample was incubated at 50°C for 1 h with amyloglucosidase (70 U) and α-amylase (4U) to hydrolyze the starch to glucose. The glucose produced by starch hydrolysis was then measured as described before by spectrophotometric coupled enzymatic assay.

### Cell Wall Components

The determination of cell wall components was performed following the procedure described in the NREL/TP-510-42619 method, with minor modifications as described in Santi et al. (2015). After the separation of the water and ethanol extractives, and gravimetric quantification (data not reported), the solid residue was hydrolyzed to determine the amount of total lignin (acid-soluble + acid-insoluble lignin), including the monosaccharides liberated by the hydrolytic process, according to the NREL/TP-510-42618 method (Santi et al., 2015). Monomeric sugars resulting from the acid hydrolysis of the extractive-free solid residue were quantified by high-performance anion-exchange chromatography with pulsed amperometric detection (HPAEC-PAD) equipped with a gold working electrode (1 mm in diameter) and an Ag/AgCl reference electrode (Dionex™ ICS-5000; Thermo Fisher Scientific, Waltham, MA, United States). An analytical CarboPac PA20 (Thermo Fisher Scientific, Waltham, MA, United States) column (3 × 150 mm) with a relative guard column was used. All runs were carried out at 30°C using a mobile phase gradient with two aqueous solutions: (A) NaOH 200 mmol L^−1^; (B) NaOH 100 mmol L^−1^ with NaAcetate 100 mmol L^−1^ at a flow rate of 0.4 ml min^−1^ as described in Giannino et al. (2020). Cellulose content was calculated from the glucose derived by acidic hydrolysis applying an anhydro correction (0.9 factor) due to the glycosidic bond cleavage. Hemicellulose content was calculated as the sum of monomers arabinose, galactose, xylose, and mannose, and the pectin content was calculated as the sum of fucose, rhamnose, and galacturonic acid.

### Antioxidants and Pigments

Total anthocyanins were extracted following the protocol described by Proietti et al. (2019). The frozen powder of rocket leaves (70 mg) was extracted in 2 ml 1% HCl in methanol for 1 h at 65°C. The liquid extract was separated by centrifugation at 16,000 × g for 5 min. After centrifugation, the supernatant was separated, and total anthocyanin content was quantified spectrophotometrically measuring the absorbance at 560 nm; concentrations were expressed as cyanidin-3-glucoside equivalent values. For pigment determination, chlorophylls (Chl a and Chl b) were quantified spectrophotometrically using the same ethanolic extracts used for NSC determination, as described in Lichtenthaler and Wellburn (1983) and following the formula: Chl a = 13.95_A665_−6.88_A649_; Chl b = 24.96_A649_−7.32_A665_, where A665 and A649 indicate the absorbance measured at 665- and 649-nm wavelength, respectively.

β-Carotene and lutein were extracted from frozen rocket leaf disks (1.8 cm^2^) with 2 ml 100% acetone at 4°C under dark conditions using a glass-glass homogenizer. The samples were centrifuged at 16,000 × g for 5 min at 4°C and filtered through a 0.2 μm nylon PPII syringe disposable filter; 15 μl of the clear extract was used to determine the concentration of pigments by an HPLC U3000 system (Dionex™ ICS-5000; Thermo Fisher Scientific, Waltham, MA, United States), equipped with a C18(2) LUNA (Phenomenex, Bologna, Italy) analytical column (5 μm, 250 mm × 4.6 mm) and a related guard column (Phenomenex, Bologna, Italy) maintained at 30°C. All separations were achieved isocratically using, from 0 to 4 min, a mobile phase composed of solution A: 1.75% water, 1.75% methanol, 1.75% dichloromethane, and 94.75% acetonitrile, and from 4.1 to 18 min a mobile phase composed of solution B: 50% acetonitrile and 50% diethyl acetate, with a final re-equilibration of 4 min with solution A. The flow rate was 1 ml min^−1^ for a total run time of 22 min. The autosampler was maintained at 4°C, the UV detector wavelength was set at 440 nm, and concentrations of carotene and lutein were determined against standard curves (Žnidarčič et al., 2011). For the determination of ascorbic acid, 60 mg of the frozen powder was extracted in a glass-glass homogenizer with 2 ml of 3% TCA at 4°C. The mixtures were centrifuged at 16,000 × g for 5 min at 4°C. The supernatants were filtrated through a 0.45-μm nylon disposable filter and used to determine the concentration of ascorbic acid using an HPLC U3000 (Dionex™ ICS-5000, Thermo Fisher Scientific, Waltham, MA, United States) system. The HPLC system was equipped with an Acclaim TM Polar Advantage C16 (Dionex™ ICS-5000; Thermo Fisher Scientific, USA) analytical column (5 μm, 250 mm × 4.6 mm) maintained at 30°C and a related guard column. The extract was eluted using 200 mM KH_2_PO_4_ (pH 2.8) as the mobile phase, at a flow rate of 0.7 ml min^−1^ under isocratic conditions. The autosampler was maintained at 4°C, and the sample loop size was 5 μl. The UV detector wavelength was set at 254 nm, and concentrations of ascorbate were determined against an ascorbic acid standard curve.

### Inorganic Anions and Organic Acids

The ethanolic extracts used for NSC determination were also used for the quantification of inorganic anions and organic acids. After centrifugation, the supernatants were filtrated through a 0.2 μm nylon PPII syringe filter prior to injection on an ion chromatography system (Dionex™ ICS-5000; Thermo Fisher Scientific, Waltham, MA, United States) equipped with a conductivity detector, an analytical IonPac AS11-HC column (4 × 250 mm) (Thermo Fisher Scientific, Waltham, MA, United States) with a related guard column and an IonPac Anion Trap Column (ATC)-1 (Thermo Fisher Scientific, Waltham, MA, United States). The system was coupled with an ERS™ 500 Electrolytically Regenerated Suppressor (Dionex™ ICS-5000; Thermo Fisher Scientific, Waltham, MA, United States). Runs were carried out at 30°C and a flow rate of 1 ml min^−1^ using a sodium hydroxide stepped gradient as reported in Proietti et al. (2019). The electrical signal was integrated into micro-Siemens (μS). The eluents and the inorganic anion and organic acid standard solutions were prepared using HPLC-grade reagents (Merck KGaA, Darmstadt, Germany). U3000-HPLC and ICS-5000 chromatography system control, data acquisition, and processing were performed with the software Chromeleon Data System 6.8 (Dionex™ ICS-5000; Thermo Fisher Scientific, Waltham, MA, United States).

### Experimental Design and Statistical Analysis

Statistical analysis was performed by one way ANOVA using the STATISTICA software package (StatSoft for Windows, 1998). When the F-test was significant, differences between averages were tested by the LSD test with *P* = 0.05.

## Results

### Growth, Yield Efficiency, and Product Characteristics

Continuous light from emergence to harvest, over a 30-day cycle, with two different light spectra and at 300 μmol m^−2^ s^−1^ of photosynthetically active radiation neither impaired rocket growth nor caused visual bolting or damage to the leaves (Figure 1). The growth and productivity of the rocket plants were significantly affected by different lighting conditions (Table 1), with those grown under W-CL treatment conditions outperforming the others in terms of production of total fresh and dry matter, and fresh and dry leaf weight. Both CL treatments had a higher leaf dry weight than the W-12h treatment. On a daily basis, the average of total fresh matter production of the treatments were 130, 188, and 145 g m^−2^ day^−1^ for W-12 h, W-CL, and RB-CL, respectively, while the total dry matter productivity was 8.7, 14.6, and 12.7 g DW m^−2^ day^−1^, with an increase of 68 and 46% for W-CL and RB-CL over W-12h respectively. Considering the carbon (C) concentration of the three treatments, and assuming the same in the roots, the average light use efficiency (μmol CO_2_ μmol^−1^ PAR) was 0.010, 0.017, and 0.015 for the three treatments, with an increase of 68 and 51% for W-CL and RB-CL, respectively, over W-12h. The average light conversion efficiency into edible produce was 0.3, 0.51, and 0.43 (μmol CO_2_ μmol^−1^ PAR day^−1^) for W-12h, W-CL, and RB-CL, respectively. DM% of the rocket leaves increased only in the RB-CL light treatment. The SLDW was 22 and 36% lower in the plants produced under W-CL and W-12h conditions, respectively, compared with the parameter measured in leaves produced under RB-CL conditions. Root fresh and dry biomass was higher in RB-CL than in the other two treatments, while root DM content was not affected by the growth light (Table 1). S/R ratio was higher in the rocket plants grown under W-CL conditions than in plants grown under W-12h and RB-CL light treatment conditions. The ash content in the aerial parts of plants grown under W-12h light treatment conditions was 5 and 17% higher than that in the aerial parts of those grown under W-CL and RB-CL treatment conditions, respectively (Table 1), but W-CL accumulated more ash per day, and unit surface, than the other two treatments. The leaf concentration of carbon and nitrogen showed a contrasting trend in the rocket plants grown under different light treatment conditions. C concentration was highest in RB-CL, and there were no statistically significant differences between W-CL and W-12h. N concentration showed the highest value in the rocket leaves grown under the W-12h light treatment conditions, and decreased by 21 and 26% in the rocket leaves grown under W-CL and RB-CL light treatment conditions, respectively. C/N ratio was highest in the leaves grown under RB-CL light treatment conditions, while the lowest value was observed for the W-12h light treatment (Table 1). The protein concentration was higher in the W-12h light treatment than in W-CL (−18%) and W-CL (−23%); however, protein yield was higher in the W-CLlight treatment than in W-12h (−31%) and RB-CL (−21%).

**Table 1:** Growth parameters and composition of the aerial part of *Eruca vesicaria* (L.) Cav. plants grown under three different light treatment conditions.

**Light treatment**			
**Growth parameters**	**W-12 h**	**W-CL**	**RB-CL**
Total fresh matter (g FW · m^−2^)	3890 ± 241 **b**	5639 ± 214 **a**	4336 ± 244 **b**
Total dry matter (g DW · m^−2^)	261.6 ± 5.3 **c**	438.4 ± 17.1 **a**	382.0 ± 21.3 **b**
Leaf (g FW · m^−2^)	3375 ± 156 **b**	5087 ± 210 **a**	3390 ± 216 **b**
Leaf(g DW · m^−2^)	232.5 ± 5.0 **c**	397.3 ± 16.7 **a**	318.7 ± 22.1 **b**
DM%	6.89 ± 0.39 **b**	7.81 ± 0.20 **b**	9.40 ± 0.30 **a**
SLDW (g of leaf · m^−2^)	43.9 ± 2.20 **c**	56.2 ± 4.47 **b**	68.7 ± 4.19 **a**
LAI	5.29 ± 0.11 b	7.10 ± 0.29 a	4.64 ± 0.32 b
Root (g FW · m^−2^)	515.0 ± 90.0 **b**	551.8 ± 6.1 **b**	946.8 ± 62.3 **a**
Root (g DW · m^−2^)	31.1 ± 3.20**b**	38.0 ± 0.60**b**	49.6 ± 5.0 **a**
DM%	6.25 ± 0.46	6.88 ± 0.09	6.22 ± 0.12
shoot/root	7.48 ± 0.72 **b**	10.50 ± 0.41 **a**	6.43 ± 0.27 **b**
Ash (%DW)	16.0 ± 0.06 **a**	15.3 ± 0.17 **b**	13.7 ± 0.09 **c**
Ash (g m^−2^ day^−1^)	1.24 ± 0.01**c**	2.02 ± 0.02**a**	1.44 ± 0.01**b**
C (% DW)	35.3 ± 0.54**b**	35.5 ± 0.10**ab**	36.5 ± 0.29**a**
N (%DW)	5.30 ± 0.05**a**	4.20 ± 0.03**b**	3.90 ± 0.14**c**
C/N	6.65 ± 0.14**c**	8.46 ± 0.08**b**	9.40 ± 0.32**a**
Protein (% DM)	23.6 ± 0.35 **a**	19.7 ± 0.33 **b**	18.7 ± 0.08 **c**
Protein (g m^−2^)	54.4 ± 0.79**b**	78.2 ± 3.07**a**	62.0 ± 4.05**b**

*Values are expressed as mean ± standard error (n = 6). Mean values with different letters are statistically different (P = 0.05)*.

### Non-structural Carbohydrates

Rocket tissues accumulated relevant amounts of NSC that were more abundant in the shoot than in the root, 3 and 0.4%, respectively, of the fresh weight on average of all treatments (Tables 2, 3). In the leaves, starch was the most abundant NSC followed by glucose, which was several times higher than sucrose and fructose. Starch and glucose, together, accounted for more than 75% of the NSCs in leaves. However, the plants grown under different light regime conditions had significantly different NSCs, with differences being more pronounced in the shoots than in the roots. In the shoots, starch increased drastically under both CL treatment conditions (97 and 126% higher in W-CL and RB-CL with respect to W-12h, respectively) and was also shown by the starch/total soluble NSC ratio, which increased from 0.76 in W-12h to 1.2 and 1.3 in the W-CL and RB-CL treatments, respectively. Under CL treatment conditions, on average, the rocket leaves accumulated 20% more glucose than the leaves grown under W-12h light treatment conditions. On average, soluble sugars increased by 26% in CL with respect to the 12-h photoperiod, while the total NSC in the RB-CL and W-CL treatments increased by 53 and 72% with respect to W-12h growth light conditions, respectively (Table 2). In the roots, NSCs were about 10 times less than in the leaves, with sucrose being the main NSC (Table 3). Glucose and fructose contents were significantly higher in the roots of the W-CL grown plants (Table 3); moreover, sucrose content was statistically lower in the RB-CL treatment than in either W-12h or W-CL treatment. Starch content in the roots was very low and not affected by the light treatments. Total carbohydrate content was mainly affected by the content of soluble carbohydrates in the roots and was statistically higher in the roots grown under W-CL conditions, with a decrease of about 18 and 39% seen in the W-12h and RB-CL treatments, respectively.

**Table 2:** Non-structural carbohydrate (NSC, as mg · 100 g FW^−1^) content in the leaves of *Eruca vesicaria* (L.) Cav. grown under three different light treatment conditions.

**Light treatment**			
**NSC**	**W-12h**	**W-CL**	**RB-CL**
Glucose	375.0 ± 26.5**b**	458.9 ± 16.1**a**	471.1 ± 18.9 **a**
Fructose	71.6 ± 6.18 **b**	94.6 ± 2.95 **a**	76.1 ± 8.30 **b**
Sucrose	70.5 ± 3.58 **b**	78.9 ± 4.19 **b**	126.9 ± 5.13 **a**
Total soluble	517.1 ± 32.7**b**	632.4 ± 20.5**a**	674.1 ± 29.1 **a**
Starch	394.7 ± 31.7**b**	776.8 ± 36.0**a**	893.3 ± 53.7**a**
Starch/total soluble	0.76 ± 0.04**b**	1.23 ± 0.06 **a**	1.33 ± 0.11 **a**
Total Carbohydrate	911.8 ± 57.1**c**	1409.2 ± 44.9**b**	1567.4 ± 56.3**a**

*Values are expressed as mean ± standard error (n = 6). Mean values with different letters are statistically different (P = 0.05)*.

**Table 3:** Non-structural carbohydrate (NSC, mg · 100 g FW^−1^) content in the roots of *Eruca vesicaria* (L.) Cav. grown under three different light treatment conditions.

**Light treatment**			
**NSC**	**W-12h**	**W-CL**	**RB-CL**
Glucose	30.3 ± 2.24 **b**	72.3 ± 3.75 **a**	38.8 ± 2.26 **b**
Fructose	8.9 ± 0.65 **b**	11.1 ± 0.52 **a**	7.5 ± 0.76 **b**
Sucrose	95.0 ± 3.74 **a**	82.8 ± 5.38 **a**	49.6 ± 4.25 **b**
Total Soluble	134.2 ± 6.6 **b**	166.2 ± 6.5 **a**	95.9 ± 5.3 **c**
Starch	9.1 ± 0.90	8.7 ± 0.48	10.6 ± 1.38
Total Carbohydrate	143.3 ± 7.4 **b**	174.9 ± 6.4 **a**	106.5 ± 6.4 **c**

*Values are expressed as mean ± SE (n = 6). Mean values with different letters are statistically different (P = 0.05)*.

### Cell Wall Components

The light treatments significantly affected the contents of the cell wall components measured (lignin) and estimated by the content of the prevailing monomers (cellulose, hemicelluloses, and pectin) (Table 4A). The content of cellulose was about five times higher than that of hemicellulose and lignin, and two-and-a half times higher than that of pectins (fucose + rhamnose + galacturonic acid). Cellulose showed a significantly higher content in the plants grown under RB-CL treatment conditions. Hemicellulose content was similar in the leaves grown with RB-CL and W-12h but decreased with the W-CL treatment. Lignin amounts were lowest in the rocket plants grown under RB-CL treatment conditions. Monosaccharide components of hemicellulose, galactose, arabinose, xylose, and mannose were affected by all the light treatments; in particular, arabinose content was 16% lower in the leaves grown with W-CL and W-12 h as compared with the RB-CL treatment. Galactose, xylose, and mannose amounts were statistically similar in the rocket leaves from the RB-CL and W-12h treatments, while decreasing in averages, respectively, of 14, 31, and 51% in the plants under W-CL conditions (Table 4A). A statistically significant difference in pectin content was recorded only between W-12h and W-CL, with the former showing a 13% higher content than the latter. Among the monomers, the only rhamnose was significantly lower in W-CL than in the other two treatments (Table 4A). The total fiber composition of the aerial parts of the rocket plants showed a reduction in the amount in the W-CL treatment with respect to the other two. On a dry weight basis, the calculated cell wall components accounted for 57% of the DW in the W-12h treatment and 43% of the DM in both CL treatments (Table 4B). The decrease in cell wall components due to CL was higher for those with a secondary prebiotic role (hemicellulose + pectins, 27% with respect to W-12h on average) than for those recalcitrant to the gut microbiota fermentation (cellulose + lignin, 20% with respect to W-12h on average).

**Table 4A:** Cell wall component (% fresh weight) content in the leaves of *Eruca vesicaria* (L.) Cav. grown under three different light treatment conditions.

**Light treatment**			
**Cell wall components(% FW)**	**W-12h**	**W-CL**	**RB-CL**
Cellulose	2.06 ± 0.126 **b**	1.80 ± 0.041 **b**	2.42 ± 0.035 **a**
Total lignin	0.481 ± 0.016 **a**	0.449 ± 0.037 **ab**	0.390 ± 0.014 **b**
Hemicellulose	0.448 ± 0.019 **a**	0.360 ± 0.012 **b**	0.492 ± 0.004 **a**
Arabinose	0.115 ± 0.003 **b**	0.111 ± 0.001 **b**	0.135 ± 0.001 **a**
Galactose	0.137 ± 0.004 **a**	0.121 ± 0.004 **b**	0.142 ± 0.002 **a**
Xylose	0.137 ± 0.008 **a**	0.096 ± 0.004 **b**	0.141 ± 0.001 **a**
Mannose	0.059 ± 0.011 **a**	0.032 ± 0.003 **b**	0.074 ± 0.001 **a**
Pectins	0.83 ± 0.03 **a**	0.72 ± 0.03 **b**	0.76 ± 0.03 **ab**
Fucose	0.012 ± 0.001	0.011 ± 0.001	0.012 ± 0.0004
Rhamnose	0.058 ± 0.002 **a**	0.047 ± 0.002 **b**	0.054 ± 0.0004 **a**
Galacturonic acid	0.764 ± 0.030	0.665 ± 0.028	0.696 ± 0.029
Total fibers	3.83 ± 0.10 **a**	3.34 ± 0.12 **b**	4.07 ± 0.05 **a**

*Values are expressed as mean ± standard error (n = 6). Mean values with different letters are statistically different (P = 0.05)*.

**Table 4B:** Cell wall component (% dry matter) content in the leaves of *Eruca vesicaria* (L.) Cav. grown under three different light treatment conditions.

	**Light treatment**		
**Cell wall components (% DM)**	**W-12h**	**W-CL**	**RB-CL**
Hemicell + Pectins	18.6± 0.25 **a**	13.9± 0.55 **b**	13.4± 0.33 **b**
Cellulose + Total Lignin	36.9 ± 1.62 **a**	28.9 ± 0.97**b**	29.9 ± 0.25 **b**
Total fibers	55.5 ± 1.48**a**	42.7 ± 1.52 **b**	43.2 ± 0.58**b**

*Values are expressed as mean ± standard error (n = 6). Mean values with different letters are statistically different (P = 0.05)*.

### Antioxidants and Pigments

Total anthocyanins were measurable only in the CL treatments, so a statistical analysis (one way ANOVA) was performed only for the values obtained under W-CL and RB-CL conditions (Table 5). The highest amount of ascorbic acid in the rocket leaves was measured in plants grown under W-CL light treatment conditions (Table 5), with a significant increase of about 8% with respect to the content measured in W-12h light treatment; the leaves grown under RB-CL conditions did not show significant changes compared with the other two light treatments (Table 5). Contents of the different pigments in the rocket leaves were significantly affected by the light treatments (Table 5). Total chlorophyll (Chl a + Chl b) was lower when RB-CL was applied during growth, compared with the values measured in the leaves grown under W-CL and W-12h treatment conditions. The carotenes were similarly higher in the rocket leaves grown under RB-CL and W-12h lighting conditions, while their values halved in the leaves grown under the W-CLtreatment conditions (Table 5). Lutein showed highest value in the rocket leaves grown under RB-CL treatment conditions, while the content of this pigment was similar in leaves grown under W-CL and W-12h treatment conditions.

**Table 5:** Antioxidant content in the leaves of *Eruca vesicaria* (L.) Cav. grown under three different light treatment conditions.

**Light treatment**			
**Antioxidant parameters**	**W-12h**	**W-CL**	**RB-CL**
Anthocyanins	–	1,21 ± 0,17 **ab**	2,18 ± 0,24 **a**
Ascorbic acid	96.4± 4.73 **b**	104.4 ± 1.51 **a**	99.6 ± 1.35 **ab**
Chl (a + b)	41.6 ± 1.21 **a**	43.4± 1.54 **a**	31.9± 1.20 **b**
Carotenes	4.02 ± 0.25 **a**	2.52 ± 0.09 **b**	3.94 ± 0.17 **a**
Lutein	12.6 ± 0.74 **b**	13.1 ± 2.57 **b**	44.3 ± 5.21 **a**

*Total anthocyanins are reported as cyanidin-3-glucoside equivalent (mg Cy3-GE ·100g FW^−1^); ascorbic acid, chlorophylls, total carotenes, and lutein contents are reported as mg ·100g FW^−1^. Values are expressed as mean ± standard error (n = 6). Mean values with different letters are statistically different (P = 0.05)*.

### Inorganic Anions and Organic Acids

Nitrate content was significantly reduced in the rocket plants grown under W-CL and RB-CL conditions compared with the plants grown with the photoperiod of 12 h dark/light (W-12h) (Table 6). The reduction was 22 and 16% under W-CL and RB-CL treatment conditions, respectively. Nitrate measured in leaves grown under W-CL and RB-CL treatments were statistically similar. Malic acid content was about 10 times higher than citric acid level and showed an increase in rocket leaves under RB-CL of 41 and 19% when compared with the W-12h and W-CL treatments, respectively. Citric acid level did not show any statistical differences among the light treatments applied (Table 6).

**Table 6:** Nitrate (mg · kg FW^−1^ or ppm) and organic acid (mg · 100 g FW^−1^) content in the leaves of *Eruca vesicaria* (L.) Cav. grown under three different light treatment conditions.

**Light treatment**			
	**W-12h**	**W-CL**	**RB-CL**
**Inorganicanion**			
Nitrate	4594 ± 185 **a**	3582 ± 211 **b**	3849 ± 135 **b**
**Organic acid**			
Malic acid	201 ± 6.0 **b**	278 ± 13.0 **ab**	343 ± 16.0 **a**
Citric acid	18.0 ± 3.0	21.0 ± 1.0	28.0 ± 5.0

*Values are expressed as mean ± standard error (n = 6). Mean values with different letters are statistically different (P = 0.05)*.

## Discussion

### Yield and Growth Characteristics

Rocket yield was high under the tested conditions for all the light treatments. The amount of fresh produce obtained in our system was higher when compared with the yield recorded for rockets under the previous field and controlled environment conditions (Jones-Baumgardt et al., 2020). A higher yield was, however, reported when the rocket was cultivated under high natural irradiance conditions in floating systems (Petropoulos et al., 2016), with presumably much higher daily light integral (DLI) than the one used in our experiments. Our results confirm a great potential for high-yield rocket production under soilless systems and controlled growth conditions (Fontana and Nicola, 2009), and show that rocket can be a good candidate species for space-related cultivation (Colla et al., 2007; Dueck et al., 2016; Chandler et al., 2020) and vertical farming initiatives, where plants are grown uniquely with artificial lighting.

In *E. vesicaria*, CL increased the growth and productive performances of plants with minor effects depending on the wavelength applied; furthermore, it did not cause visual bolting or damage to leaves. Our activity was part of the EDEN ISS project, and our growing conditions were similar to those used by Zabel et al. (2020) during the overwintering growing period in Antarctica. In the experiments reported here, the yield of both CL treatments was higher than that obtained by Zabel et al. (2020). This is particularly evident considering that rockets grown in the EDEN ISS project had 25% more DLI than those in this experiment. The higher yield in our case could be due to the higher plant density and growing temperature. This emphasizes the importance of a complete evaluation of all growing parameters to maximize yield under CEA conditions. Gitelson et al. (1976) tested wheat and several vegetable species under fully controlled growth conditions and continuous light, and obtained very high yields for wheat, while yields of several leafy vegetable species were compared with those obtained with a rocket in this experiment, even if they used a much higher DLI. Our light conversion into edible biomass was similar to the highest conversion values obtained recently by Li et al. (2019) in space-oriented experiments with several vegetable species grown under fully controlled conditions and no CL. The positive results obtained in the rocket plants when CL was applied together with white light (W-CL) are in agreement with the data reported for different plant species (Gitelson et al., 1976; Lin et al., 2013). Conversely, in other experiments, contradictory effects of CL on growth, yield, and DM production were obtained. These were ascribed to the interactive effects of CL with light intensity, plant species, and different growth conditions that were applied (Sysoeva et al., 2010). Lighting extended to 24 h was ineffective for DM accumulation in lettuce, cucumber, corn, and radish, while a decrease in growth and yield was observed in tomato and sweet pepper (Murage and Masuda, 1997). A higher increase in DM accumulation was obtained in lettuce, radish, and roses when exposed to a low PPFD for a long photoperiod, rather than when plants were exposed to a higher PPFD for a short day length under the same daily integrated PPFD (Sysoeva et al., 2010). This agreed with the trend of DM accumulation observed in *E. vesicaria* grown under 300 PPFD in CL (W-CL and RB-CL), when compared with the growth under 600 PPFD applied for 12 h (W-12h). Growth under CL has effects on many aspects of plant growth and development, whose interplay with the genetic bases of each species is largely unclear. The higher productivity and the inferred photosynthetic assimilation rate per unit of growing area, measured in our CL treatments, support the hypothesis that CL caused an increase in assimilating availability. This is in accordance with the strong increase in the NSC content of the leaves. An increase in photosynthetic efficiency in CL treatments can be explained by the canonical increase in photosynthetic efficiency with the decrease in light intensity. In relation to leaf anatomy, growth in CL can stimulate both cell extension and division with decreasing leaf thickness and number of cell layers (Hay and Heide, 1983), and consequently a lower SLDW. We cannot exclude that a faster increase of leaf area in the early phases of plant growth could have contributed to the overall increase in biomass accumulation. At harvest, however, SLDW was higher in plants grown under CL, especially under RB-CL treatment conditions, which is in agreement with a higher DM value and with a higher percentage of carbon (C%). Growth at low light intensity is well known to cause a reduction in proteins per unit of leaf area (Terashima and Evans, 1988). Accordingly, a decrease in N% and protein content occurred under CL conditions. In our case, however, this result could also be ascribed to the dilution effects caused by faster growth and the accumulation of NSCs per unit of leaf area. The decrease of proteins per unit of leaf area under low light is normally linked to a rearrangement of the photosynthetic apparatus that leads to a decreased assimilation rate and, thus, growth (Terashima and Evans, 1988). This was not the case in the CL grown plants, showing that the decrease in light intensity was more than compensated by the longer photoperiod. The ash percentage reported in the literature for *E. vesicaria* ranged from 9.5 to 19.3% of dry matter (Bukhsh et al., 2007; Nurzyńska-Wierdak, 2015). The ash percentage measured in the rocket leaves was close to the highest level of this range, with a lower percentage observed in the leaves grown under CL than under W-12h conditions, suggesting that in the rocket plants grown under CL conditions, the mineral content was diluted by the higher biomass production. Moreover, the value of ash was related to that of plant transpiration, which in plant leaves showed a linear increase with an increase in PPFD (Barman et al., 2008).

### Non-structural Carbohydrates

The accumulation of NSCs in the shoots grown under CL conditions is in line with the evidence that CL increased photosynthetic product availability, as demonstrated by the higher yield of CL vs. 12h light growth conditions, and which was reported in previous studies on tomato and pepper (Dorais et al., 1996), and on eggplant (Murage and Masuda, 1997). Photosynthesis must be coordinated for end-product synthesis (sucrose in the cytoplasm and starch in the chloroplast) and export (normally in the form of sucrose) to avoid the accumulation of photosynthetic end-product-induced feedback inhibition of photosynthesis. CL, therefore, by eliminating the diurnal alternation between photosynthate production in the light and their use in the dark, can have a detrimental effect on the source-sink equilibrium. Indeed, Dorais et al. (1996) ascribed the carbohydrate accumulation under continuous light to the onset of leaf injuries and lower plant production in tomato and sweet pepper. In the case of the rocket, however, our results show that, under the DLI used, there was no general negative effect on the balance between photosynthesis and photosynthate utilization by plant sinks in CL. A direct evaluation of the effect of DLI on the structural characteristic of the photosynthetic apparatus and on its performances *in vivo* could be of interest in future studies.

The light recipe affected the use efficiency of extra photosynthate produced under CL conditions. RB-CL produced more dry matter than W-12h but less than W-CL. The higher starch and sucrose levels in the shoot and the lower levels in the root of RB-CL in comparison with W-CL and a related data set (DM% in leaves, SLDW, Leaf area index, root growth, shoot/root ratio) indicate that plants under RB-CL had more difficulties than the W-CL in using the extra photosynthetic product derived by CL. The limitation was not due to limited root growth; hence, it was not linked to reduced root sink capacity. An effect of the spectrum recipe on the shoot sink capacity, mainly due to LAI formation, could explain this evidence. It is well known that the relative abundance of different light wavelengths can affect shoot morphogenesis. Our data indicate that under CL, the presence of a substantial amount of green, some far red, and a red/blue ratio close to one, favored photosynthetic product utilization more than the RB-CL recipe with prevailing R wavelength. The lower LAI of RB-CL than that of W-CL points to a limit for leaf growth and expansion, possibly linked to the absence of far red light in this treatment (Legendre and van Iersel, 2021). This evidence indicates that while a CL light setup is not detrimental to yield in *E.vesicaria*, performances can be better modulated by optimization of the light recipe, which deserves more research.

When dealing with produce quality, high NSC content in leaves should be considered a positive quality trait. High total soluble NSC content can have a positive impact on the taste of the produce. Moreover, soluble sugars are compatible with osmolytes, and their abundance might help contrasting water loss and to wilt in the post-harvest period, and might increase the shelf life, crispness, and palatability of the produce. Finally, high NSC, caused by ensuring abundant respiratory substrates may substantially increase the shelf life of the produce. CL had a positive effect on quality aspects related to the content of NSC.

Non-structural carbohydrates are nutrients available for all humans. The nutritionally available fraction of food from plants should be considered a key quality aspect and a variable when evaluating the efficiency of the production system, in addition to the harvest index. CL then not only increased the light conversion efficiency into total and shoot biomass, but it also increased the efficiency of NSC production, thereby increasing the efficiency of light conversion for the nutritionally relevant fraction of the produce. This aspect is still scarcely considered in terrestrial open field food production systems, but it should be of primary importance when dealing with high energy-intensive systems, such as vertical farming and bioregenerative life support systems for space. The importance of this aspect is immediately evident while designing bioregenerative life support systems for space where its two-fold effect of increasing nutrient production efficiency and reducing the amount of human solid excreta needing recycling should be implemented in the system design.

### Fibers

This describes for the first time the estimated quantity, composition, and monomeric components of the cell wall in *E. vesicaria* produce in relation to growth under specific light conditions. Cell wall components are a relevant fraction of most plant tissues at the vegetative stage when no significant accumulation of storage compounds has occurred. They might also represent a major fraction of dietary fiber in plant food (Elleuch et al., 2011; Pastell et al., 2019; Giannino et al., 2020), playing a key role in human well-being (Kaur et al., 2021). The definition and measuring methods of food fiber have changed in time depending, more so, on the regulatory body involved. The method most referred to, and widely accepted by regulatory bodies for food fiber determination, is based on sequential enzymatic attack and separation steps that fractionate the fiber into defined amounts (Elleuch et al., 2011). Rising evidence indicates that the plant fiber present in food has a relevant role in the functionality of the gut microbiome and its health promoting role in many organisms, including humans (Kaur et al., 2021). A chemical characterization of fiber in terms of the type of compound and monomeric composition is necessary to elucidate the link between food fiber type and its effect on the gut microbiome (Kaur et al., 2021). The value of fiber content estimated in this study, based mostly on chemical analysis of monomeric components of the cell wall, is higher than the values reported in the literature. This could indeed depend on the measuring method and on the growing conditions and cropping system. In rocket leaves, fibers such as cellulose, hemicellulose, and lignin represented about 79% of the total fiber content, while pectins represented about 21%. In the rocket leaves, the cellulose, representing about 60% of the total fiber, was positively affected by CL and specifically by the RB-CL treatment, confirming that higher photoassimilate availability under CL plant growth conditions could be redirected to cell wall building (Verbančič et al., 2018) while also indicating a potential role of red light photoreceptors in cellulose synthase complex (CESA) activation (Fankhauser and Staiger, 2002). In hypocotyls of *Arabidopsis* grown in the dark, Bischoff et al. (2011) showed that the inhibition of the CESA complex and its effects were reversed *via* phyB activation. Light also affects lignin deposition, Dong et al. (2015) showed that the extension of light cycle reduced lignin contents in wheat, which correlates with the results obtained here for rocket leaves. Hemicelluloses and pectin are synthesized and deposited into the apoplast for assembly into the cell wall. In the rocket leaves, pectin content was substantially unaffected by the light treatments, while hemicellulose content showed a lower value in W-CL and tendentially the higher value of the RB-CL treatment. The changes in pectin, in particular hemicellulose levels, could correlate the increase in *E. vesicaria* plants grown with the mechanical properties of the cell wall. Miyamoto et al. (1994) showed that in the Avena coleoptile, a decrease in cell wall polysaccharides and consequent reduced extensibility of the cell wall, resulted in decreased growth.

### Antioxidants and Pigments

Continuous lighting significantly increases photo-oxidative pressure that results in the accumulation of antioxidant bioactive compounds (Yabuta et al., 2007). Anthocyanin levels and ascorbic acid concentration have been influenced by light quality applied during plant growth. The effect of blue and red radiation in increasing anthocyanin concentration was reported in numerous studies (Li and Kubota, 2009; Samuoliene et al., 2012; Nicole et al., 2016; Thoma et al., 2020). Moreover, a long photoperiod and low light intensity (18 h and 200 PPFD) increased the anthocyanin index in Red Oak and Red Salanova lettuce (Nicole et al., 2016). The anthocyanin content measured in rocket leaves agreed with the published data but only for the rocket leaves grown under CL (W-CL, RB-CL) conditions, with the value doubling in the presence of RB radiation. Ascorbic acid levels reached in rocket leaves had the highest value under W-CL conditions; however, the response of ascorbic acid to lighting regimes can differ with respect to the plant species considered. The application of blue light, or a combination of red and blue light, significantly increased ascorbic acid content in lettuce leaves and Chinese cabbage (*Brassica campestris* L.) (Ohashi-Kaneko et al., 2007; Li et al., 2012), and in Arabidopsis grown under CL conditions, a significant accumulation of ascorbic acid was found (Yabuta et al., 2007). The role of anthocyanins and ascorbic acid in scavenging free radicals in biological systems defines these metabolites as essential compounds for human health, protecting the human organism against reactive oxygen species, and could be particularly relevant in space food systems to counteract the detrimental effects of radiation on both plants and humans.

The effects of light on leaf pigment amounts were wide-ranging, showing positive or negative responses depending on photoperiod (Lefsrud et al., 2006; Stutte et al., 2009; Sysoeva et al., 2010; Žnidarčič et al., 2011), the wavelength applied during plant growth (Li and Kubota, 2009; Kopsell et al., 2017) and plant species (Sysoeva et al., 2010). In the rocket leaves, the amount of total chlorophylls was negatively affected when CL was applied in addition to RB radiation (RB-CL), relative to the W-12h and W-CL light treatments. β-carotene and lutein are the major carotenoids in leafy vegetables. Carotenoids in plants act as free-radical scavengers that minimize the damage of photosynthetic components, a function that is useful for human health, preventing cardiovascular diseases and thwarting cancer development (Eggersdorfer and Wyss, 2018), while lutein, in addition, protects human eyes from light-induced damage and age-related degeneration. Knowledge on the effect of CL on carotenoids is very limited in higher plants; however, differences due to cultivar variation, seasonal growth, and post-harvest conditions are known to affect their content in plants (Kamal et al., 2020). The accumulation of β-carotene and lutein was found in kale in a 24-h photoperiod (Lefsrud et al., 2006), while positive effects of blue wavelengths, *via* LED or conventional light, on the accumulation of carotenoids and xanthophylls was reported for spinach (Ohashi-Kaneko et al., 2007), lettuce (Li and Kubota, 2009; Johkan et al., 2010), Chinese kale (Kopsell et al., 2017), potato (Paradiso et al., 2019), and tropical species (Ramalho et al., 2002). In *E. vesicaria*, the lower content of chlorophylls measured in the leaves grown under RB-CL conditions has been compensated by the increase in carotenoid content. When W-CL was applied, the chlorophylls and lutein values were similar to that measured in W-12h light treatment, while β-carotene content surprisingly decreased, confirming the decisive effect of RB wavebands alone on carotenoids synthesis. Thus, light quality and extended photoperiod can be useful in improving the healthy characteristics of rocket salad by increasing the antioxidant properties and leafy pigment amount.

### Nitrate and Organic Acids

*Eruca vesicaria* is a nitrate-rich leafy vegetable, with threshold amounts of nitrate in the European Union set at 6,000 mg/kg of fresh weight in the spring-summer months and at 7,000 mg/kg of fresh weight in the autumn-winter months (Commission Regulation (EU) No 1258/2011; Siomos and Koukounaras, 2007). Indeed, despite the putative beneficial or dangerous effects of nitrate compounds on human health is currently under discussion (Habermeyer et al., 2015), nitrate intake in the human diet is associated with methaemoglobinaemia syndrome and certain forms of cancer; thus, the levels of these compounds in vegetables are still severely regulated by international authorities. Nitrate accumulates in edible tissues of plants in relation to genotypic characteristics, cultivation techniques, and growth environment conditions. Light intensity, spectral quality, and photoperiod are very important factors affecting plant nitrate levels (Proietti et al., 2013; Bian et al., 2015; Signore et al., 2020). Modification of the R:B light ratio is effective in decreasing nitrate concentration in plants, and some values are reported in relation to cultivation practices and cultivars (Qi et al., 2007; Wanlai et al., 2013). Recently, Signore et al. (2020) studied the influence of the light spectrum (full spectrum, R, B, and R + B) applied in combination with nitrogen fertilization in two rocket species, *E. vesicaria* (L.) Cav. and *Diplotaxis tenuifolia* (L.) DC. This study showed a species-specific effect of N treatment and red light spectrum on decreased nitrate concentration with higher N application, and on increased nitrate reductase activity with lower N supply. In rocket leaves, nitrate levels were lower in plants grown under CL, in accordance with the literature (Bian et al., 2015) and irrespective of the wavelength applied. Moreover, in CL, it is probable that higher carbohydrate levels found in rocket leaves may provide a carbon skeleton for nitrogen assimilation, affecting nitrate reductase at the transcription and translation levels (Bian et al., 2015). Nitrate reductase is positively regulated by light; thus, CL on rocket leaves may increase the activity of nitrate reductase and, consequently, the reduction of nitrate. Furthermore, Bliznikas et al. (2012) reported that high-soluble sugars, in particular high glucose content, can elicit the expression of the nitrate reductase gene, resulting in thereduction of nitrate level in plant tissues. In the rocket leaves, this negative correlation has been confirmed, as the higher content of glucose and soluble carbohydrates correlates negatively with the content of nitrates (respectively, R^2^ = −0.9 and R^2^ = −0.83, data not shown) in growth under CL conditions (data not shown).

The rocket leaves exposed to RB-CL displayed a higher malic acid content, and while the citric acid content showed a trend similar to that of malic acid, its value was statistically unchanged in the leaves regardless of the light treatment applied. Organic acid amount and composition can be associated with the taste and organoleptic traits of fruits and vegetables, comparable with the effects of sugar content on the sweetness or sourness of a product (Proietti et al., 2019).

In conclusion, our results suggest that CL increases the growth and quality performances of the rocket, especially in shoot tissue, representing the edible and marketable part of leafy vegetables. The enhanced improvement of extended lighting conditions on different parameters measured in the rocket is reported in Supplementary Material and expressed in g m^−2^. Furthermore, the data indicate that applying low light intensity for an extended photoperiod could be an effective way to optimize light use efficiency toward biomass production in rocket leaves without inducing injury in these plant tissues. Moreover, positive effects on the nutritional quality of rocket were observed when continuous lighting was used both alone or in synergetic application with R-B LED wavelengths, resulting in higher accumulation of NSCs and fibers in rocket leaves and improvement in antioxidant potential *via* compounds, such as ascorbic acid, anthocyanins, chlorophylls, and carotenoids. Conversely, the nitrate content, potentially harmful to human health, was significantly reduced in the rocket leaves grown under CL conditions regardless of the light spectrum applied. The results obtained for the growth and nutritional capacity of rocket salad confirm that careful scheduling of light management *via* LED lamps allows for the modulation of light spectrum, intensity, and photoperiod, thereby, enhancing plant yield and produce quality.

## Data Availability Statement

The original contributions presented in the study are included in the article/Supplementary Material, further inquiries can be directed to the corresponding authors.

## Author Contributions

AB, SP, and SM: conceptualization. SP, SM, and FR: methodology. SP, SM, AB, and PD: writing, original draft preparation, review, and editing. AB: funding acquisition. All authors have read and agreed to the published version of the manuscript.

## Funding

Support for this study was provided by: H2020 Project -EDEN ISS-Ground Demonstration of Plant Cultivation Technologies and Operation in Space for Safe Food Production on-board ISS and Future Human Space Exploration Vehicles and Planetary Outposts. Grant agreement ID: 636501; and by the project: *In-situ* Resource Bio-Utilization per il supportoalla vita nelloSpazio (ReBUS) from the Agenzia Spaziale Italiana (ASI): Prot. ASI n. 1714 del 19 feb. 2018 –n° DC-VUM-2017-080 Bando di Ricerca per missioni future di esplorazione umana dello spazio—Area tematica Sistemi Biorigenerativi.

## Conflict of Interest

The authors declare that the research was conducted in the absence of any commercial or financial relationships that could be construed as a potential conflict of interest.

## Publisher's Note

All claims expressed in this article are solely those of the authors and do not necessarily represent those of their affiliated organizations, or those of the publisher, the editors and the reviewers. Any product that may be evaluated in this article, or claim that may be made by its manufacturer, is not guaranteed or endorsed by the publisher.
