# Supplementary material for: Continuous Lighting Promotes Plant Growth, Light Conversion Efficiency, and Nutritional Quality of Eruca vesicaria (L.) Cav. in Controlled Environment With Minor Effects Due to Light Quality
Source: Front Plant Sci. 2021 Oct 12;12:730119. doi: 10.3389/fpls.2021.730119 (PMC8546256; doi:10.3389/fpls.2021.730119)
Supplement: Supplementary file 1 [file Table_1.docx]

**SUPPLEMENTARY MATERIAL**

**Table 1S**: Yield (g · m^-2^) of different components measured in fresh leaves of *Eruca vesicaria* (L.) Cav. grown under three different light treatments. Values are expressed as mean ± standard error (n = 6). Mean values with different letters are statistically different (P-value = 0.05).

|  | **Light treatment** | | |
| --- | --- | --- | --- |
| **Parameters** | **W-12h** | **W-CL** | **RB-CL** |
| **NSC** |  |  |  |
| Glucose | 12.65 ± 0.894 **c** | 23.35 ± 0.818 **a** | 15.97 ± 0.641 **b** |
| Fructose | 2.42 ± 0.209 **b** | 4.81 ± 0.150 **a** | 2.58 ± 0.281 **b** |
| Sucrose | 2.38 ± 0.121 **b** | 4.06 ± 0.213 **a** | 4.30 ± 0.174 **a** |
| Total soluble | 17.45 ± 1.105 **c** | 32.17 ± 1.041 **a** | 22.85 ± 0.99 **b** |
| Starch | 13.32 ± 1.071 **c** | 39.52 ± 1.831 **a** | 30.29 ± 1.821 **b** |
| Total carbohydrate | 30.77 ± 1.925 **c** | 71.69 ± 2.285 **a** | 53.14 ± 1.908 **b** |
|  |  |  |  |
| Ash | 37.29 ± 0.168 **c** | 60.57 ± 0.630 **a** | 43.30 ± 0.320 **b** |
|  |  |  |  |
| **Fibers** |  |  |  |
| Cellulose | 69.62 ± 4.246 **c** | 91.80 ± 2.077 **a** | 82. 03 ± 1.190 **b** |
| Total lignin | 16.23 ± 0.555 **b** | 22.86 ± 1.902 **a** | 13.24 ± 0.475 **b** |
| Hemicellulose | 15.12 ± 0.655 **b** | 18.29 ± 0.622 **a** | 16.70 ± 0.122 **ab** |
| Pectins | 28.15 ± 0.965 **b** | 36.81 ± 1.573 **a** | 25.85 ± 0.993 **b** |
| Total fibers | 129.12 ± 3.451 **b** | 169.77 ± 6.044 **a** | 137.81 ± 1.842 **b** |
|  |  |  |  |
| Protein | 54.35 ± 079 **b** | 78.18 ± 3.07 **a** | 62.00 ± 4.05 **b** |
| Nitrate | 14.84 ± 0.662 **b** | 18.23 ± 1.075 **a** | 13.05 ± 0.458 **b** |
| Malic acid | 6.79 ± 1.117 **b** | 14.16 ± 0.643 **a** | 11.63 ± 0.529 **a** |
| Citric acid | 0.59 ± 0.087 **b** | 1.07 ± 0.031 **a** | 0.96 ± 0.159 **ab** |
| Ascorbic acid | 3.25 ± 0.16 **b** | 5.32 ± 0.096 **a** | 3.38 ± 0 042 **b** |
| Chl (a+b) | 1.41 ± 0.053 **b** | 2.21 ± 0.103 **a** | 1.08 ± 0.053 **c** |
| Carotenes | 0.14 ± 0.011 | 0.13 ± 0.004 | 0.12 ± 0.020 |
| Lutein | 0.435 ± 0.03 **b** | 0.67 ± 0.131 **b** | 1.40 ± 0.187 **a** |
| Anthocyanins | - | 0.06 ± 0.009 | 0.74 ± 0.008 |
